# Expanding spontaneous pneumothorax in COVID-19 pneumonia: Case report and review of literature

**DOI:** 10.34172/jcvtr.2021.16

**Published:** 2021-02-07

**Authors:** Afshin Mohammadi, Behdad Boroofeh, Alisa Mohebbi, Mohammad Mirza-Aghazadeh-Attari

**Affiliations:** ^1^Department of Radiology, Urmia University of Medical Sciences, Urmia, Iran; ^2^Department of Internal Medicine, Urmia University of Medical Sciences, Urmia, Iran; ^3^Students’ Scientific Research Center, Tehran University of Medical Sciences, Tehran, Iran; ^4^Medical Radiation Sciences Research Center, Tabriz University of Medical Sciences, Tabriz, Iran

**Keywords:** CT, Chest, Pneumothorax, COVID-19

## Abstract

Coronavirus disease 2019 has presented itself with a variety of clinical signs and symptoms. One of these has been the accordance of spontaneous pneumothorax which in instances has caused rapid deterioration of patients. Furthermore pneumothorax may happen secondary to intubation and the resulting complications. Not enough is discussed regarding cases with COVID-19 related pneumothorax and proper management of these patients. The present article reports an elderly patient with spontaneous pneumothorax secondary to COVID-19 and reviews the existing literature.

## Introduction

The severe acute respiratory syndrome – coronavirus-2 (SARS-CoV-2) causes various clinical signs and symptoms but usually presents itself as a mild form of viral pneumonitis. Epidemiologic studies have shown that although most cases of those infected with the virus do not show severe signs or symptoms.^1^ However, pediatric and the elderly, and individuals with pre-existing conditions are at increased risk of respiratory distress syndrome, inappropriate immune responses, and other un-common manifestations of the disease.^2^ Studies focusing on those mentioned above demographic have suggested that they manifest specific radiologic signs and clinical scenarios, which can make diagnostic workup and clinical decision making complicated.

Chest CT has been one of the most prominent diagnostic tools available for clinicians, yielding a higher sensitivity than other diagnostic methods, such as molecular assays. The collective findings in chest CT of infected individuals consist of multi-focal ground-glass opacities, airspace consolidation, and air-Bronchogram. Nevertheless, other unusual imaging signs are also reported, such as nodular lesions, cavities, tree-in-bud appearances, halo and reverse halo signs, and more.^3^

One of the rarely mentioned radiologic findings has been visible visceral pleural edges and non-existent lung markings on the periphery of the lungs, which is suggestive of pneumothorax.^4^ In the present case report, we discuss an elderly male patient who developed spontaneous pneumothorax secondary to COVID-19.

## Case Presentaion

An 82-year-old male, with a history of coronary artery disease, presented to our emergency ward with low-grade fever, dyspnea and cough. The patient had a respiratory rate of 21, pulse rate of 90 and blood pressure of 135/80 on admission. Pulse oximetry revealed a blood O2 saturation of 87%. The patient did not smoke, did not have any pre-existing respiratory disease and had an active lifestyle.

The patient was then visited by an internal medicine specialist and underwent diagnostic workup for COVID-19. Lab results were otherwise typical except a mild lymphopenia (1100 /mm^3^)

**Figure 1:**
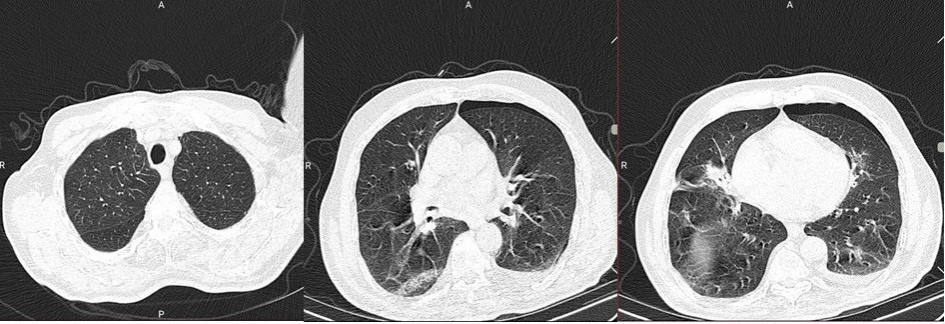
First Chest CT of the patient showing posterior-peripheral ground-glass opacities, with a thin radiolucent line presenting a small pneumothorax. The patient had minimal involvement in the apex of the lungs, and involvement was mostly observed in the bases

The initial CT scan obtained on the first day of admission (Figure 1) showed a uni-focal peripheral ground-glass opacity in the middle segments of the right lung. A small linear line of non-existent lung texture was visible on the left lung periphery. Based on institutional guidelines, the patient was admitted, and conservative management was chosen for the pneumothorax, and the initial estimation of its size was 7% of the hemi-thoracic cavity. The results of the molecular assay obtained on the first day of admission came back positive on the third day. The patient was put on hydroxychloroquine 200 twice daily, ceftriaxone 500 mg daily and Tamiflu 75 mg twice daily. He was also given steroids (prednisolone, 40 mg, IV). The patient had a stable clinical course until the fifth day of hospitalization when the patient reported an aggregation of dyspnea and emergence of dull chest pain, localized in the periphery of the left hemithorax. The patient had an ECG took, which was normal, Troponin-I was negative, Vital signs were stable, and emergency consultation with a cardiologist did not yield any further results. Physical examination yielded no positive findings other than a mildly reduced respiration sounds on the left hemithorax.

**Figure 2:**
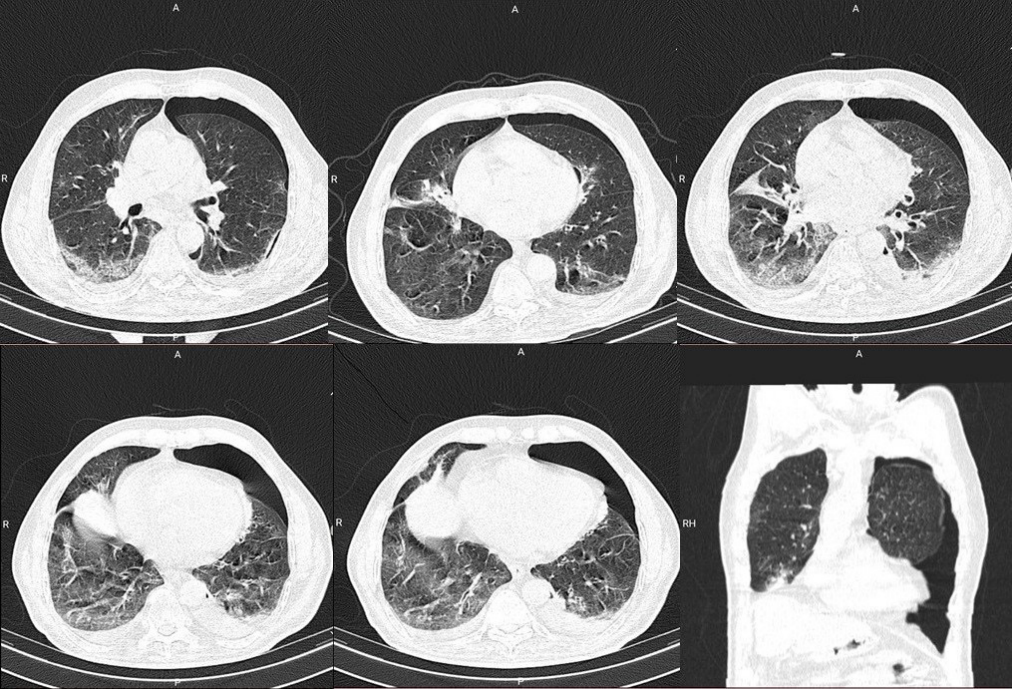
Second CT of the patient taken after a rapid deterioration in clinical signs and symptoms. The pneumothorax had extended beyond its original boundaries and occupied over one-fifth of the thoracic cavity, which warranted insertion of a chest tube. Ground-glass-opacities also increased in size and frequency over the time between the two chest CTs

The patient had a chest X-ray performed which showed signs of pneumothorax, with a barely visible reduced lung marking in the periphery of the left lung. The patient had a second CT imaging done (Figure 2), which showed a radiolucent peripheral space, a grossly visible visceral pleural edge and absent lung markings in the periphery. The diagnosis of an expanding pneumothorax was established, and utilizing the Colling’s method, the percentage of the pneumothorax was estimated to be 25.3%. The patient had an urgent consultation done with the pulmonology department, which recommended that a chest tube be inserted. The patient was stable after insertion of the chest tube, and had a complete absorption of the pneumothorax, and was discharged six days after the insertion of the chest tube.

## Discussion

Early studies being reported from china showed that most patients infected with the novel coronavirus had conventional signs of viral pneumonia. However, as the virus spread, more scholars pointed out the rare complication of the infection, such as neurovascular and dermatologic involvement, and significant involvement of the respiratory tract. Probably the two most important of these complications have been pulmonary thromboembolism and pneumothorax. These phenomena can complicate a patient with pre-existing respiratory compromise, and as they may mimic clinical signs of the coronavirus, timely diagnosis may prove to be a challenge.

Until the day this manuscript is being written, a limited number of pneumothoraxes have been reported in the setting of COVID-19. Based on aetiology, pneumothorax can be classified into two main groups, spontaneous and Iatrogenic/traumatic. The first group can be further classified to primary and secondary spontaneous. Primary spontaneous pneumothorax happens in individuals with no pre-disposing lung condition, and secondary spontaneous pneumothorax is defined as having a pneumothorax secondary to specific pathologies of the respiratory system, such as the existence of bullae, emphysema, etc. scholars suggest that COVID-19 can cause pneumothorax in several ways. It can cause excessive coughing, which can cause pneumothorax, and also can directly invade the alveoli, induce inflammation and damage the delicate respiratory epithelium. COVID-19 can form cystic lesions, increasing the risk of pneumothorax.^5^

A review of the literature revealed 18 discrete cases of pneumothorax in the setting of COVID-19 (Table 1). Most of the patients were elderly male with pre-existing conditions, with left-sided or bilateral pneumothorax. Older women were less involved. Importantly, a substantial number of young adults existed among the patients. Most of the authors declared that they treated their patients with chest tube insertion. This could have resulted from delayed diagnosis, as the clinical picture may be blurred by COVID-19. We also report an elderly patient infected with COVID-19 who had a pneumothorax and possibly misdiagnosed in the early stages. Any rapid deterioration of COVID-19 infected individuals should prompt a diagnostic workup towards pulmonary thromboembolism and pneumothorax. Clinical studies show that detecting pneumothorax in the elderly may be a challenge, as it is less symptomatic, may not be associated with pleuritic chest pain. Furthermore, a pneumothorax may have deleterious effects in the elderly compared to younger patients.^20^

**Table 1:** List of previous case reports of COVID-19 complicated with pneumothorax

**Reference**	**Post Intubation**	**History of trauma**	**Gender**	**Age**	**Pre-existing conditions**	**Initial presentation on admission**	**Side**	**Treatment**	**Other Radiologic signs**
^5^	No	No	Male	24	None	Yes	Left	tube thoracostomy	GGO
^6^	No	Yes (falling on right side)	Male	55	None	No	Bilateral	Chest tube insertion	GGO, Consolidation
^7^	No	No	Male	62	None	No	Right	Conservative	GGO, pneumomediastinum,
^3^	No	No	Male	38	binaural hearing loss and tinnitus	No	Left	Conservative	GGO, consolidation, mediastinal emphysema, giant bulla,
^8^	No	No	Male	36	10 p/y smoking, childhood asthma	Yes	Left	emergency needle decompression and then chest tube insertion	GGO
^4^	No	No	Male	26	None	Yes	Right	Chest tube insertion	Collapse
^9^	No	No	Male	38	Heavy smoker, excess alcohol consumption	No	Left	Conservative	GGO, consolidation
^10^	Yes	No	Male	70	N/A	No	Left	Chest tube insertion,,Video-assisted thoracoscopic surgery)	GGO
^10^	Yes	No	Male	56	Heavy smoker,	No	Left	Video-assisted thoracoscopic surgery following a failed chest tube	GGO
^11^	No	No	Female	82	No	Yes	Left	Chest tube insertion	pneumomediastinum, left-sided massive pneumothorax and subcutaneous emphysema
^12^	No	No	Male	87	COPD	Yes	Left	Chest tube insertion	Collapse, GGO, consolidation
^13^	No	No	Male	67	N/A	Yes	Bilateral	Chest tube insertion	GGO, pneumomediastinum
^13^	No	No	Female	84	prosthetic valve replacement, renal failure, Heart failure, hypertension, hypercholesterolemia	No	Bilateral	N/A	GGO, pneumomediastinum
^14^	Yes	No	Male	59	decompensated cirrhosis, liver transplantation	No	N/A	Chest tube insertion	GGO
^15^	Yes	No	Female	59	Morbid obesity	No	Right	Surgical intervention	GGO, pneumomediastinum
^16^	Yes	No	Male	67	coronary artery bypass, tuberculosis, chronic bronchitis, and emphysema	No	Bilateral	Chest tube insertion	Subcutaneous emphysema, mediastinal emphysema, GGO
^17^	Yes	No	Male	31	smoker	No	N/A	N/A	GGO
^18^	yes	No	Female	70	none	No	N/A	N/A	GGO
^19^	No	No	Male	41	None	Yes	Left	Chest tube insertion	GGO, pneumomediastinum. subcutaneous emphysema

Of interest, pneumothorax has been seen in the setting of COVID-19 in neonates delivered from infected mothers. Although the relationship is not defined, and the occurrence of pneumothorax could be related to prenatal complications.^21^

Currently, contradicting evidence exists regarding the proper management of a symptom-free pneumothorax, but some studies do show conservative management to be non-inferior to invasive interventions.^22^ The limited evidence existent in regards to COVID-19 associated pneumonia favor the early utilization of more invasive methods.

## Conclusion

COVID-19 associated spontaneous pneumothorax may have an inclination to progress and cause severe deterioration. Prompt management with chest tube insertion seems to be superior to conservative management, especially in high-risk demographic groups such as the elderly.

## Competing interests

The authors declare no conflict of interest.

## Ethical approval

This study was approved by the local ethics committee of Urmia University of Medical Sciences. The patient had signed a written informed consent note.
